# Fruit Quality and Metabolomic Analyses of Fresh Food Accessions Provide Insights into the Key Carbohydrate Metabolism in Blueberry

**DOI:** 10.3390/plants12183200

**Published:** 2023-09-07

**Authors:** Chunhong Zhang, Jie Li, Jialuan Wang, Lianfei Lyu, Wenlong Wu, Weilin Li, Yaqiong Wu

**Affiliations:** 1 Jiangsu Key Laboratory for the Research and Utilization of Plant Resources, Institute of Botany, Jiangsu Province and Chinese Academy of Sciences (Nanjing Botanical Garden Mem. Sun Yat-Sen), Qian Hu Hou Cun No. 1, Nanjing 210014, China; chzhang@cnbg.net (C.Z.); nj_lijie@foxmail.com (J.L.); wjl163youxiang@163.com (J.W.); njbglq@163.com (L.L.); 2 Co-Innovation Center for Sustainable Forestry in Southern China, Nanjing Forestry University, 159 Longpan Road, Nanjing 210037, China; wlli@njfu.edu.cn

**Keywords:** blueberry (*Vaccinium corymbosum* L.), flavor profile, fresh food quality, sugar metabolism, LC-MS/MS

## Abstract

Blueberry is a nutrient-rich berry, and its taste and flavor directly determine the consumer preference. Until now, few studies have focused on the comparison of fresh food quality and the key metabolites in superior fresh-eating blueberry cultivars. Herein, fruit quality indicators of 10 highbush blueberry cultivars were evaluated using ‘Bluerain’ as the control. Appearance quality analysis of fruits showed that ‘Brigitta’ had a larger fruit size and ‘Anna’ was the smallest. ‘Anna’ fruits, followed by ‘O′Neal’, had the highest ratio of soluble solids to acidity because of their lowest titratable acidity content. Despite the high soluble sugar content, the antioxidants in ‘Anna’ fruits such as total flavonoids, anthocyanins and vitamin C were lowest among all cultivars, while ‘Duke’ seemed to have opposite patterns. Furthermore, a total of 553 and 557 metabolites were identified by non-targeted metabolomics liquid chromatography-tandem mass spectrometry (LC-MS/MS) in positive and negative ion mode, respectively. Particularly, the numbers of differentially accumulated metabolites (DAMs) were the most between the ‘O′Neal’ vs. ‘Bluerain’ group. The DAMs involved in the metabolic pathways, sesquiterpenoid and triterpenoid biosynthesis, monoterpenoid biosynthesis, galactose metabolism, starch and sucrose metabolism, may be mainly related to the synthesis of flavor and carbohydrate substances. Moreover, the expression patterns of genes involved in sugar metabolism were verified by quantitative real-time PCR (qRT-PCR) analysis in different cultivars. Therefore, the systematical comparison of the quality characteristics, metabolites and expression profiles of related genes in highbush blueberries with good flavor could provide some basis for further research on fresh fruit breeding of blueberries.

## 1. Introduction

Blueberry is a high-value berry and evergreen shrub of the genus *Vaccinium* L. in the *Ericaceae* family [1]. It has attracted great interest over recent years for the beneficial antioxidant properties of its bioactive compounds and desirable flavor worldwide [2]. Three main species of blueberries, including wild lowbush, highbush and rabbiteye blueberries are differentially marketed as different species. Highbush blueberry fruits are larger and have more pulp, less skin and lower anthocyanins, total phenolics and antioxidant capacity than wild lowbush blueberry fruits [3]. The highbush blueberry fruit is pivotally marketed as fresh fruit and half of them are frozen for later marketing or processing [4,5].

Identifying consumer blueberry preferences is only the first step in creating better fresh fruit cultivars. The future success of many fruit crops relies on their ability to satisfy consumer preferences. The flavor of blueberry fruit products is an important quality parameter that influences consumer satisfaction and resulting demand [6]. Breeding for cultivars with good flavor is also challenging as blueberry biochemistry compounds that have a high degree of genetic control are the main determinants of fruit taste [7]. The chemical composition that contributes to the unique blueberry flavor includes sugars, acids and volatile compounds. Sweet and sour are both primary tastes [8]. Olfaction is the sense of smell and flavor, and flavor is the sense of compounds during mastication, called retronasal olfaction [9]. Significant positive correlations to the overall liking of blueberry fruit were found with sweetness, texture and flavor in proper order, while sourness had a significantly negative relationship with overall liking [7].

Many of the compounds affecting consumer preference for blueberries, such as fructose, pH, β-caryophyllene oxide and 2-heptanone, have been sufficiently genetically controlled [7]. A variety of volatiles from various biosynthetic pathways has contributed to different blueberry aromas, such as fruity, floral, peachy or grassy-green [10]. The biochemical components in fruit that contribute to taste and flavor are mainly subject to different effects of plant genetics, including different cultivars [11]. Lowbush fruit volatiles were composed of 48% esters, 29% aldehydes and 4% monterpenoids compared to 48% aldehydes, 26% monoterpenoids and 3% esters in highbush fruit [12]. They also differentiated genotypes with floral and sweet aroma notes and confirmed that such characteristics are preferred by consumers [12]. Identifying compounds the production of which is more determined by genotypes than other effects is preferable for breeders. Not only in terms of the soluble solids content (SSC) and titratable acidity (TA), but the volatile levels also vary between *Vaccinium* species and cultivars [5,13,14]. The similar compounds in sugars and volatiles that exhibit preference in different genetic backgrounds may be promising in finding blueberry germplasm.

During fruit ripening, organic acids content decreases and sugar content increases [15]. The balance of sweetness and acidity plays essential roles in determining the fruit flavor and fruit quality [16,17]. Compared to the organic acid, contents and composition of sugars (carbohydrates), the reducing sugars glucose and fructose in blueberry were of central importance for the sweetness and taste of fruits [16,18]. Metabolites and the expression of associated genes of the synthesis of sugars to determine the sweetness in blueberries have been extensively investigated. During sugar metabolism, starch, sucrose and reducing sugars are the main compounds, and several key associated enzyme encoding genes are involved in this process. Enzymes including sucrose synthase (SS), sucrose phosphate synthase (SPS), invertase (INV) or neutral invertase (NIN) [19], β-amylase (BAM) [20] and sugar transporter proteins, such as the tonoplast monosaccharide transporter (TMT) [21,22], are all involved in the ripening and accumulation of sugars [21,23,24,25]. Expression patterns in the synthesis or transport of sugars have revealed dynamics in different blueberry cultivars, ripening stages and storage conditions [26]. Exogenous ethylene treatments accelerate blueberry softening and promote sucrose metabolism by inhibiting the expression of *VcSPS1* and *VcNIN2*, and stimulating the expression of *VcSS1* [27]. The sweeter taste of blueberries is due to down-regulated *VcINV*, *VcSPS* and aldose reductase (*VcADR*) transcription and increased sucrose concentration [28]. These data provide more proof regarding the elucidation of the mechanism of sugar accumulation in blueberries.

Blueberries have experienced extraordinary growth in consumption over the past decade. In an effort to more effectively probe into the perceived flavor components of fruit quality, a better understanding of differences in the flavor profile of blueberry species is needed. Until now, most previous studies have focused on the flavor metabolites in blueberry fruits, applying the gas chromatography-mass spectrometry (GC-MS) approach with the advantages of a high-resolution and unified database. There have been very few studies on the volatile or nonvolatile compounds in blueberries through the application of liquid chromatography-tandem mass spectrometry (LC-MS/MS), which shows a predominance in terms of its wide applicability and high sensitivity. Blueberries, especially the highbush type, have become increasingly popular as part of a healthy diet in China. However, the individual differences in various highbush blueberries with good flavor grown at the same sites have rarely been revealed. The aim of this study was to comparatively explore the common or specific fruit qualities and flavor compounds of highbush blueberry cultivars of excellent flavor as a means to support future breeding work. Therefore, as ‘Bluerain’ is a small-sized fruit with a thick peel and weaker fresh food properties [29], the ripened fruits of 10 highbush blueberry cultivars with better flavor in southern China were compared using ‘Bluerain’ as the control to identify consistent metabolites contributing to the flavors that are vital to the sensory experience. To accomplish this, the appearance quality traits and flavor quality traits of 11 blueberry cultivars were evaluated for biochemical variation. After metabolic profiling identification using LC-MS/MS, three blueberry cultivars with distinct traits were selected for further differentially accumulated metabolite (DAM) analysis, allowing for a comparison of the sugar substances and expression profiles of sugar metabolism genes. Revealing the characteristics of the flavor components of these cultivars could help us in the future utilization and breeding of flavorful blueberries.

## 2. Results

### 2.1. Appearance Quality Characteristics of Different Blueberry Fruits

Ten blueberry cultivars with a pleasant taste, using ‘Bluerain’ as the control, were analyzed to examine the appearance quality traits (Table 1). For fruit transverse diameter or longitudinal diameter, ‘Brigitta’ and ‘Springhigh’ both had an apparently larger size, while they were both smaller for ‘Anna’. As for the fruit shape index, ‘Bluerain’ and ‘Anna’ ranked first and second, respectively. ‘Brigitta’ and ‘Springhigh’ also had a higher average fruit weight among all cultivars, while ‘Bluerain’ and ‘Anna’ belonged to the small fruit type. Despite this, most of the ripened fruits exhibited good firmness above 3 N, while others such as ‘Darrow’, ‘O’Neal’, ‘Springhigh’ and ‘Duke’ were relatively softer.

### 2.2. Flavor Quality Traits of Different Blueberry Fruits

Fruit quality parameters of ten highbush blueberry cultivars of good taste were further identified (Table 2). The results showed that the soluble sugar content of ‘Anna’ was the highest (172.01 mg/g), followed by ‘Bluerain’ (151.45 mg/g) and ‘O’Neal’ (143.50 mg/g), while ‘Duke’ (115.58 mg/kg) and ‘Brigitta’ (120.84 mg/kg) showed the lowest content. The soluble solids (SS) content was similar to the distribution of the soluble sugar content in different cultivars, but ‘Bluerain’ was higher (18.42%), followed by ‘Anna’ (16.50%) and ‘O’Neal’ (16.16%). ‘Bluerain’ (1.07%) and ‘Darrow’ (1.03%) ranked first and second, respectively, in titratable acidity (TA) content, while ‘Anna’ (0.26%) and ‘O’Neal’ (0.27%) had similarly lower levels. The SS/TA ratio of ‘Anna’ (63.92) and ‘O’Neal’ (60.96) were apparently higher than those of other varieties. The following were ‘Springhigh’ (33.96) and ‘Camellia’ (31.84), while the ratios of ‘Darrow’, ‘Brigitta’, ‘Bluerain’ and ‘Zhongzhi 3’ were 14.71, 15.96, 17.24 and 17.76, respectively, indicating that their sweetness was at a regular level.

The anthocyanin content of ‘Duke’ was 2.22 mg/g, which was the highest, along with the highest content of total polyphenols and Vitamin C (VC) amongst all samples, while they were lowest in ‘Anna’. Therefore, in terms of sweetness, acidity and the solid-acid ratio, ‘Anna’ and ‘O’Neal’, had a similar higher soluble sugar content and lower titratable acidity content, which demonstrated a larger SS/TA ratio and excellent taste. Specifically, there were differences between the two. ‘Anna’ had a lower level of antioxidants in almost all varieties, while ‘O’Neal’ had a higher accumulation of anthocyanins and total flavonoids content. ‘Bluerain’ had both higher soluble sugar and titratable acidity content, which caused a low SS/TA ratio and general taste. ‘Duke’ had the lowest soluble sugar content and moderate titratable acid content, making the SS/TA ratio moderate. ‘Duke’ showed excellent performance in terms of antioxidant levels, showing a somewhat opposite accumulation trend in sugar content and antioxidant indices, while ‘Anna’ was also the same. 

In addition, there was a higher content of titratable acidity and VC in ‘Darrow’, but it also had the lowest SS/TA ratio’. The total polyphenol and soluble sugar contents were both the lowest in ‘Brigitta’. ‘Springhigh’ had a relatively lower content of total flavonoids, while ‘Zhongzhi 3’ had a lower content of soluble solids and VC. ‘Misty’ had a higher level of total polyphenols and ‘Camellia’ had a higher level in terms of the SS/TA ratio, anthocyanins and polyphenols. ‘Star’ had relatively lower contents of soluble sugar, anthocyanin, polyphenol and VC among all of the cultivars.

### 2.3. Non-targeted Metabolomics Annotation Revealed the Allocation of Metabolites

**Figure 1:**
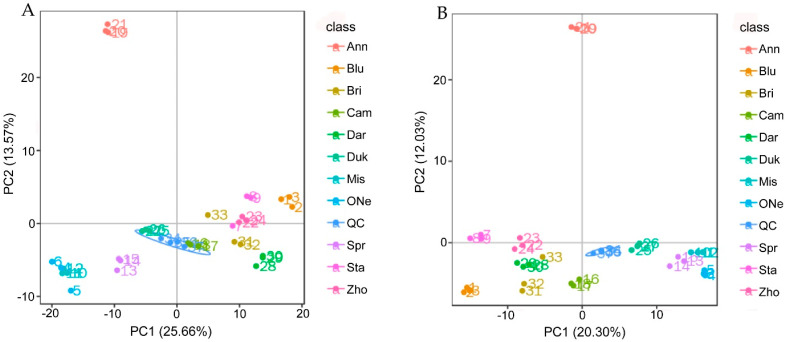
Principal component analysis (PCA) score plot of 11 blueberry samples in positive (**A**) and negative (**B**) ion modes. Ann, ‘Anna’; Blu, ‘Bluerain’; Bri, ‘Brigitta’; Cam, ‘Camellia’; Dar, ‘Darrow’; Duk, ‘Duke’; Mis, ‘Misty’; ONe, ‘O’Neal’; QC, quality control; Spr, ‘Springhigh’; Sta, ‘Star’; Zho, ‘Zhongzhi 3’.

Non-targeted metabolomic analysis was performed to further understand the metabolite composition and the flavor traits of the ten blueberry cultivars. Principal component analysis (PCA) was performed on the metabolite peaks detected in the 11 sample groups and quality control (QC) groups. The first principal component (PC1) and the second principal component (PC2) both explained the fine clustering of the metabolite information under positive (Figure 1A) and negative (Figure 1B) ion mode.

**Figure 2:**
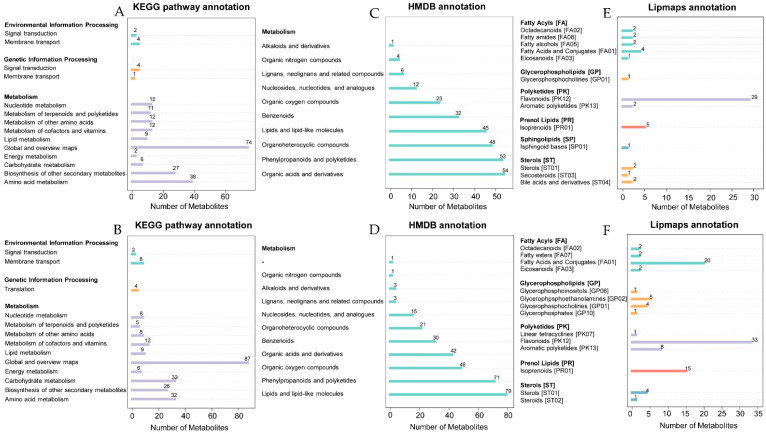
Pathway annotation and classification of metabolites in different databases of KEGG, HMDB and LIPID MAPS. (**A**) Metabolites annotated in the KEGG database in positive ion mode; (**B**) Metabolites in the KEGG database in negative ion mode; (**C**) Metabolites annotated in the HMDB database in positive ion mode; (**D**) Metabolites annotated in the HMDB database in negative ion mode; (**E**) Metabolites annotated in the LIPID MAPS database in positive ion mode; (**F**) Metabolites annotated in the LIPID MAPS database in positive ion mode. The bars in different color in (**A**,**B**) showed the category of metabolic pathways annotated in the KEGG database, and those in (**E**,**F**) showed the lipid category annotations in the LIPID MAPS database.

A total of 1110 substance peaks (553 positive ion peaks and 557 negative ion peaks) were detected from all fruits by non-targeted LC-MS/MS (Tables S3 and S4). All metabolites were annotated and classified in the database of the Kyoto Encyclopedia of Genes and Genomes (KEGG), Human Metabolome Database (HMDB), Lipid Metabolites and Pathways Strategy (LIPID MAPS). Through KEGG pathway annotation (Figure 2A,B), the metabolites were classified into three categories: Environmental Information Processing, Genetic Information Processing and Metabolism. In particular, the ‘Metabolism’ category covered a large part in terms of the number of metabolites. Under negative ion mode, the metabolites ranked in the top three were Global and overview maps (87), Carbohydrate metabolism (32) and Amino acid metabolism (32). Under positive ion mode, the metabolites ranked in the top three were Global and overview maps (74), Amino acid metabolism (38) and Biosynthesis of other secondary metabolites (27). Through HMDB classification and annotation (Figure 2C,D), the metabolites of the top three subclasses under negative ion mode were Lipids and lipid-like molecules (79), Phenylpropanoids and polyketides (71) and Organic oxygen compounds (48), while they were Organic acids and derivatives (54), Phenylpropanoids and polyketides (53) and organoheterocyclic compounds (48) under positive ion mode. The metabolites were divided into five or six categories through LIPID MAPS annotation (Figure 2E,F) under negative or positive ion mode. The different modes indicated three main classes, including Flavonoids, Isoprenoids and Fatty Acids and Conjugates.

**Figure 3:**
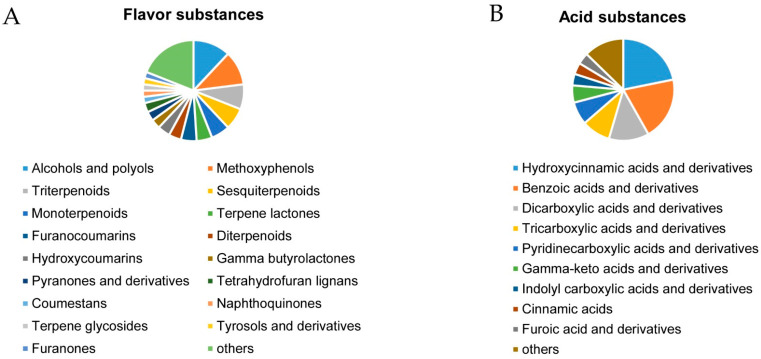
Classification map of metabolites of flavor substances (**A**) and acid substances (**B**).

The substance components of metabolites related to the fresh food quality of 11 blueberry cultivars were further analyzed. According to the annotation of subclasses under both negative and positive modes, the metabolites belonging to the flavor substances (Figure 3A) and acid substances (Figure 3B) in ripened blueberry fruits were analyzed in detail, as the total 48 sugar substances were all in the subclass of carbohydrates and carbohydrate conjugates. For flavor substances (Figure 3A), we found a total of seventeen types of metabolites with a number over two, and the other nineteen only had one type each. Among them, the numbers of alcohols and polyols (12), methoxyphenols (11), triterpenoids (8), sesquiterpenoids (7) and monoterpenoids (6) ranked in the top five types, and thus, may be the main flavor substances. The metabolites of acid substances included nine types with a number over two, and the other seven included one each (Figure 3B). The main types of acid substances were hydroxycinnamic acids and derivatives (12), benzoic acids and derivatives (11), dicarboxylic acids and derivatives (7), tricarboxylic acids and derivatives (5) and pyridinecarboxylic acids and derivatives (4).

### 2.4. Differentially Accumulated Metabolites (DAMs) Analysis

**Figure 4:**
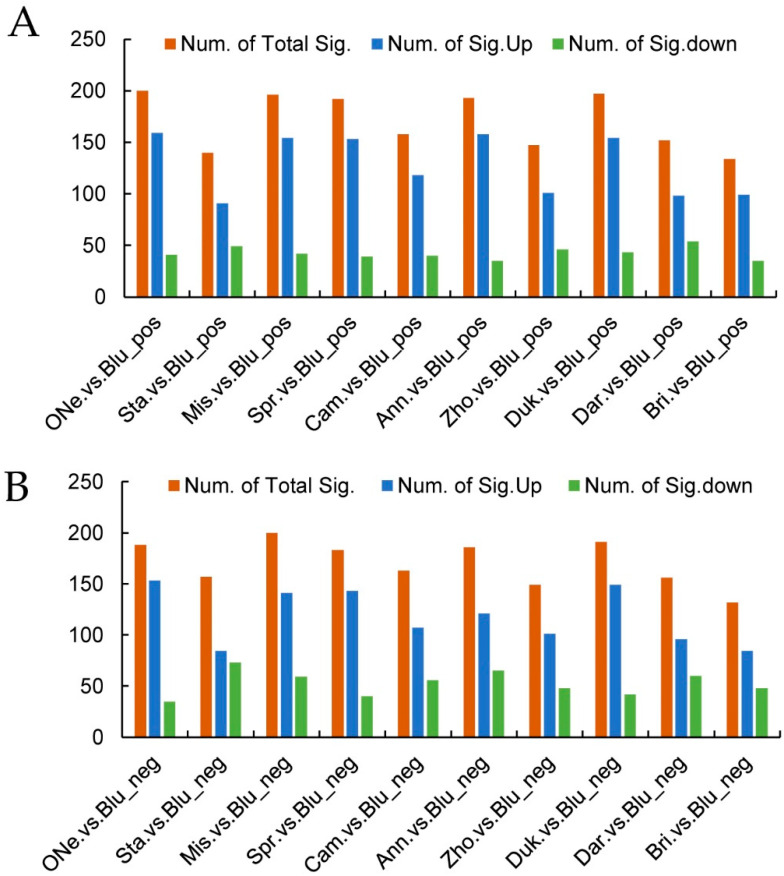
Statistics of amounts of DAMs in positive (**A**) and negative (**B**) ion modes.

DAMs analysis was further performed using ‘Bluerain’ as the control according to the variable importance in projection (VIP), fold change (FC) and *p*-value parameters (Figure 4A,B). Under positive mode, the highest number of DAMs was in the ONe vs. Blu group (200), followed by the Duk vs. Blu group (197), the Mis vs. Blu group (196), the Ann vs. Blu group (193) and the Spr vs. Blu group (192). All of these groups uniformly exhibited a significantly higher number of upregulated metabolites than downregulated metabolites. The top two amounts of upregulated metabolites were in the ONe vs. Blu group (159) and the Ann vs. Blu group (158), respectively. On the other hand, the Bri vs. Blu group (134), the Sta vs. Blu group (140) and the Zho vs. Blu group (147) showed lower amounts of DAMs. Under negative mode, the top five amounts of DAMs and upregulated metabolites were similar to the positive mode (Figure 4B), except that the highest was in the Mis vs. Blu group (200).

### 2.5. Analysis of Differentially Accumulated Carbohydrate Profiles

**Figure 5:**
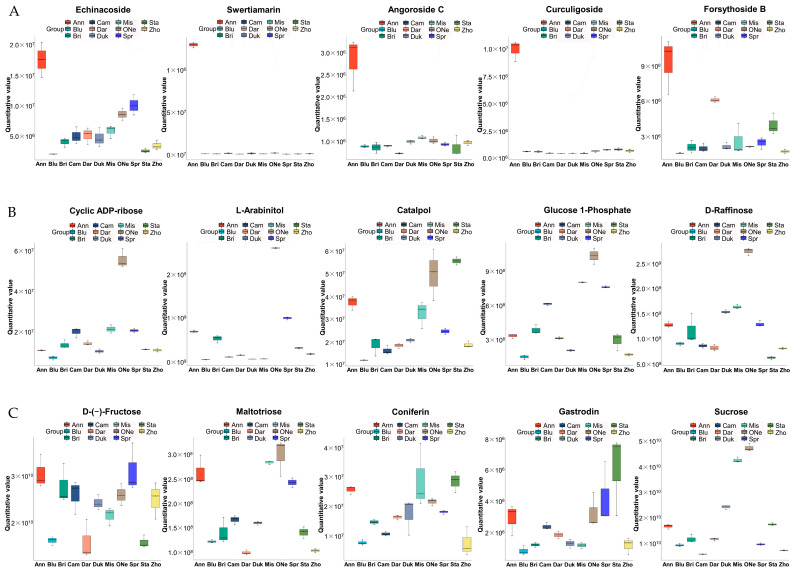
Violin plot of sugar substances dominantly accumulated in the blueberry cultivar ‘Anna’ (**A**), ‘O’Neal’ (**B**) and both cultivars (**C**).

As the two blueberry cultivars ‘Anna’ and ‘O’Neal’ both showed an obviously higher sugar content and higher SS/TA ratios, the accumulation of carbohydrates in them was analyzed and compared with ‘Bluerain’. Under negative and positive ion modes, in ‘Anna’ there were fifteen metabolites upregulated and one downregulated (nystose), which were differentially abundant and belonged to the subclass of carbohydrates and carbohydrate conjugates. Among these, the top five metabolites with higher log2FC values in comparison to ‘Bluerain’ was Echinacoside (2.18-fold, 1.78 × 10^7^), Swertiamarin (2.11-fold, 1.31 × 10^8^), Angoroside C (2.05-fold, 2.85 × 10^6^), Curculigoside (2.02-fold, 1.04 × 10^7^) and Forsythoside B (1.97-fold, 9.19 × 10^6^) (Figure 5A). D-(−)-Fructose in ‘Anna’ (3.05 × 10^10^), as a main sugar substance, also had a 1.47-fold upregulation in the ‘Anna’ vs. ‘Bluerain’ group. As for ‘O’Neal’, there were nineteen metabolites of carbohydrates and carbohydrate conjugates upregulated and two (nystose and muramic acid) downregulated. Among them, the top five with a higher log2FC value was Cyclic ADP-ribose (2.36-fold, 5.57 × 10^7^), L-Arabinitol (1.96-fold, 2.58 × 10^7^), Catalpol (1.79-fold, 4.96 × 10^7^), Glucose 1-phosphate (1.72-fold, 1.02 × 10^9^) and D-Raffinose (1.67-fold, 2.73 × 10^9^), successively (Figure 5B). Moreover, Sucrose (4.71 × 10^10^), D-(+)-Maltose (3.10 × 10^9^) and D-(−)-Fructose (2.59 × 10^10^) also showed higher levels in ‘O’Neal’ compared with ‘Bluerain’. Despite these distinctions, higher levels of seven types of carbohydrates were similarly upregulated in two cultivars (Figure 5C), including Echinacoside, L-Arabinitol, Catalpol, D-(−)-Fructose, Maltotriose, Coniferin and Gastrodin. Sucrose was specifically upregulated in the ‘O’Neal’ vs. ‘Bluerain’ group but not in the ‘Anna’ vs. ‘Bluerain’ group. Though no differences were detected for the D-(+)-Glucose metabolite in different comparison groups, a higher level (1.23 × 10^10^) was still found in ‘Anna’ compared to ‘O’Neal’ or ‘Bluerain’.

### 2.6. KEGG Pathway Enrichment Analysis of DAMs

KEGG is a powerful tool for metabolic analysis and metabolic network research to identify pathways that are enriched in differential metabolites compared to all identified metabolite backgrounds. Pathway enrichment can identify the major biochemical and signal transduction pathways involved in differential metabolites. Based on the above enrichment results, a bubble map of the enriched KEGG pathway was drawn (only the results of the top 20 are shown).

**Figure 6:**
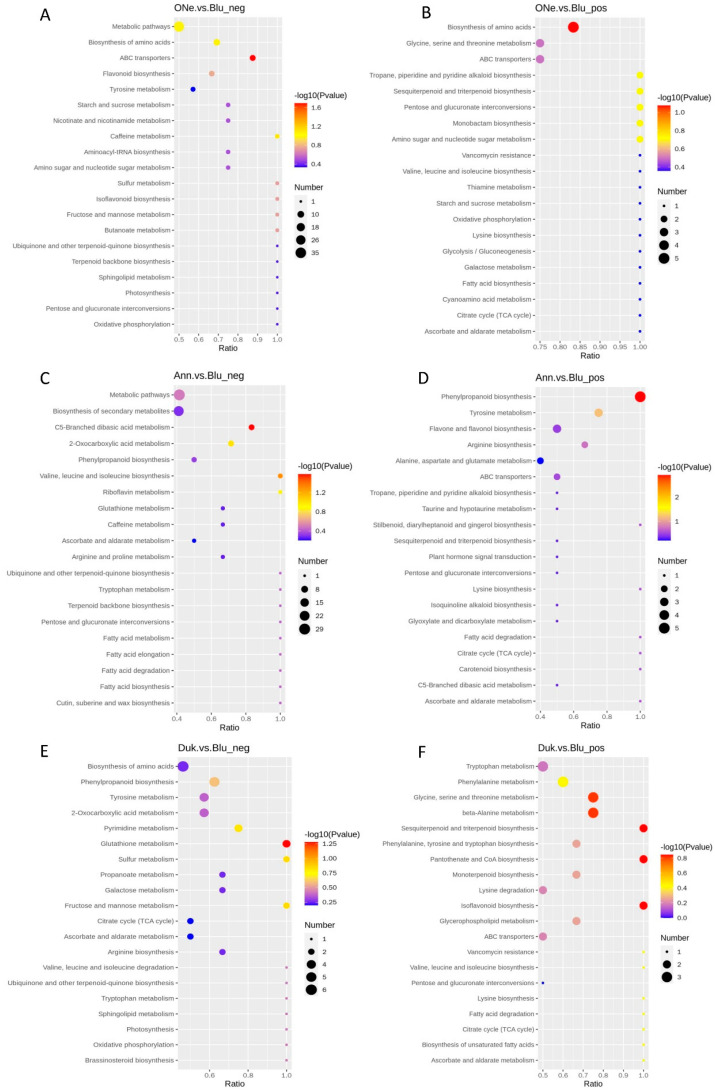
The top 20 pathways by KEGG pathway enrichment analysis based on differentially accumulated metabolites. (**A**,**B**): Pathways in ONe vs. Blu in positive and negative mode, respectively; (**C**,**D**): Pathways in Ann vs. Blu in positive and negative mode, respectively; (**E**,**F**): Pathways in Duk vs. Blu in positive and negative mode, respectively.

For the ONe vs. Blu group (Figure 6A,B), the top 20 pathways mainly included metabolic pathways, the biosynthesis of amino acids, ABC transporters, etc. There were also some related to flavor substances such as sesquiterpenoid and triterpenoid biosynthesis, terpenoid backbone biosynthesis and ubiquinone and other terpenoid-quinone biosyntheses, which were also found in the Ann vs. Blu group. We found the most pathways regarding the metabolites of carbohydrates, including starch and sucrose metabolism, fructose and mannose metabolism, amino sugar and nucleotide sugar metabolism and galactose metabolism. Comparatively, the three pathways in the Ann vs. Blu group were focused on the metabolic pathways, phenylpropanoid biosynthesis and the biosynthesis of secondary metabolites (Figure 6C,D). As for the Duk vs. Blu group, the metabolites were mainly involved in the biosynthesis of amino acids and metabolism of many kinds of amino acids (Figure 6E,F). Furthermore, we also found the pathways of galactose metabolism, fructose and mannose metabolism and monoterpenoid biosynthesis, which may be resources of flavor or carbohydrate substances in the Duk vs. Blu group. 

### 2.7. Verification of Sugar Metabolism Genes by Quantitative Real-Time PCR (qRT-PCR)

**Figure 7:**
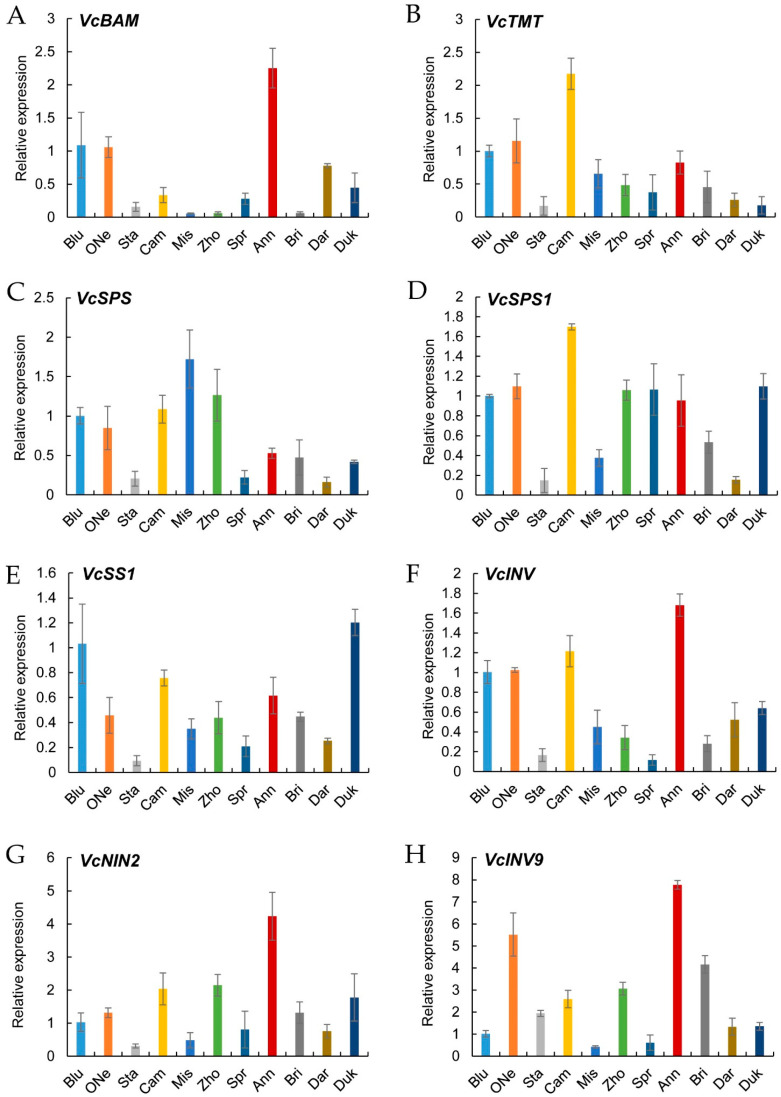
Expression patterns of eight sugar accumulation genes in eleven blueberry cultivars. (**A**) *VcBAM*; (**B**) *VcTMT*; (**C**) *VcSPS*; (**D**) *VcSPS1*; (**E**) *VcSS1*; (**F**) *VcINV*; (**G**) *VcNIN2*; (**H**) *VcINV9*.

As sweet taste is an important sensory quality standard, the expression levels of eight reported key genes associated with sugar accumulation were detected in these blueberry cultivars. The sugar metabolism genes, including one β-amylase gene (*VcBAM*), one tonoplast monosaccharide transporter gene (*VcTMT*), two SPS genes (*VcSPS*, *VcSPS1*), one SS gene (*VcSS1*) and three INV genes (*VcINV*, *VcNIN2*, *VcINV9*), were selected for qRT-PCR analysis. The expression results showed that *VcBAM* was highly expressed in ‘Anna’, ‘Bluerain’ and ‘O’Neal’ (Figure 7A). *VcTMT* showed a relatively higher level in ‘Camilia’, ‘O’Neal’, ‘Bluerain’ and ‘Anna’ (Figure 7B). We found higher levels of *VcSPS* in ‘Misty’ and ‘Zhongzhi 3’, while we found lower levels in ‘O’Neal’ and ‘Anna’ (Figure 7C). For *VcSPS1*, relatively higher levels were found in ‘Camilia’, ‘Duke’ and ‘O’Neal’, while the expression of *VcSS1* was higher in ‘Bluerain’ and ‘Duke’ (Figure 7D,E). The expression of *VcINV* was somewhat similar to *VcBAM* and showed an obviously higher level in ‘Anna’ (Figure 7F). Furthermore, we also found a higher expression level of *VcNIN2* in ‘Anna’, and *VcINV9* in ‘Anna’ and ‘O’Neal’.

## 3. Discussion

Blueberries (*Vaccinium* L.) are a widely consumed fruit worldwide, not only owing to their richness in terms of bioactive compounds but also due to their excellent sensory quality. The cultivars with these two advantages are undoubtedly the most popular. Currently, the flavor of blueberry fruits is an important parameter that determines consumer satisfaction. In this study, we compared the fruit quality traits of 11 blueberry cultivars with relatively better sensory flavor. Appearance quality showed that ‘Anna’ and the control cultivar ‘Bluerain’ were both small-sized fruits, with fruit transdiameters below 14 mm, and both had larger fruit shape indexes. ‘Springhigh’ and ‘Brigitta’ were both larger fruit types, with a transdiameter of over 17 mm, and had heavier fruit weights. Others were medium to large fruits with a fruit weight ranging from 1.47 g (‘O’Neal’) to 2.06 g (‘Duke’). Fruit firmness seemed to have no obvious relation to the fruit size as the different sizes of fruits all showed lower softness.

Titratable acidity (TA) decreased, while total soluble solids (TSS), the TSS/TA ratio and vitamin C all increased as the fruit ripened [30]. With fruit ripening, all blueberry fruits perceived optimal sensory qualities, including sweetness, acidity and aroma. Amongst them, ‘Anna’ occupied the lowest TA and vitamin C content, but had the highest soluble sugar content and TSS/TA ratio, which showed that ‘Anna’ had an extremely strong ability in terms of sugar biosynthesis. For flavor quality traits, we found that the SS/TA ratios of ‘Anna’ (63.92) and ‘O’Neal’ (60.96) were especially higher than those of other varieties because of their lower titratable acidity content, indicating their excellent taste. ‘Anna’ showed a lower quality performance amongst all cultivars, except for its excellent sweetness. In addition to sweetness performance, ‘O’Neal’ also had a higher accumulation of anthocyanins and total flavonoid content. Notably, ‘Duke’ had a relatively lower soluble solid content, and its antioxidant quality seemed to be higher due to its outstanding content of anthocyanin, total polyphenols and Vitamin C. Therefore, these three cultivars, ‘Anna’, ‘Duke’ and ‘O’Neal’, seemed to be differentially specific types corresponding to the accumulation of sugars, antioxidants and a variety of both. The accumulation of sugars and antioxidants seems to have an opposite preference, which was reflected in the ‘Anna’ and ‘Duke’ fruits. A previous study has shown that sugar and acid metabolism and flavonoid synthesis in blueberry fruits are regulated by development and genotype [31].

The targeted metabolomics of diverse blueberry accessions have shown that flavor compounds, including sugars, acids and volatiles, contribute the most to each flavor attribute [32]. A group of eight terpenoid volatiles constitutes the primary metabolic group associated with aroma sensations in blueberry fruits [33]. In the present study, we carried out a non-targeted metabolomic analysis of LC-MS/MS to detect the flavor substances of ten cultivars using ‘Bluerain’ as the control. From the overall annotation results, a number of metabolites were mainly focused on the carbohydrate metabolism and biosynthesis of other secondary metabolites under negative and positive ion modes, respectively. As for the fresh food quality, the flavor substances of 11 blueberry fruits mainly included 36 types of metabolites and the number of alcohols and polyols, methoxyphenols, triterpenoids, sesquiterpenoids and monoterpenoids ranked in the top five. This result was partially consistent with a previous study in the highbush blueberry, which indicated that aldehydes and monoterpenoids were the main volatiles in highbush blueberry fruits [12]. Furthermore, a total of twenty-eight volatiles, including five esters, eleven terpenoids, three aldehydes, six alcohols and three volatile phenols, were identified from eight blueberry fruits and distinct varietal volatile profiles existed [34]. Furthermore, fruit aromatic substances showed significant differences among wild and cultivated blueberry species and between varieties of the same species [35]. With the fruit maturation of blueberry fruits, anthocyanins, nutrients, antioxidant activity, taste and aroma all increased [30]. For acid substances, the main subclasses included hydroxycinnamic acids and derivatives, benzoic acids and derivatives, dicarboxylic acids and derivatives, tricarboxylic acids and derivatives and pyridinecarboxylic acids and derivatives. Sugar substances uniformly belonged to the subclass of carbohydrates and carbohydrate conjugates. At this point, the acidity metabolism pathways in blueberry fruits exhibited diversification, while those of sugar metabolism were relatively concentrated.

Due to the excellent sweetness of both the ‘Anna’ and ‘O’Neal’ fruits, the differentially accumulated carbohydrate profiles in these two varieties were especially elucidated. Nystose was the only common carbohydrate downregulated in both ‘Anna’ and ‘O’Neal’, which might play a certain role in maintaining the quality of the fruits during storage [36]. Moreover, the number of metabolites of carbohydrates and carbohydrate conjugates upregulated were 15 and 19 in ‘Anna’ and ‘O’Neal’, respectively. Furthermore, the top five DAMs of carbohydrates were somewhat different in these two cultivars. D-(−)-Fructose seems to have an obvious upregulation in ‘Anna’ vs. ‘Bluerain’, while Sucrose, D-(+)-Maltose and D-(−)-Fructose all demonstrated relatively higher levels in ‘O’Neal’ vs. ‘Bluerain’. Fruit sweetness was generally determined by the levels of soluble sugars, including sucrose, fructose and glucose [6]. In blueberries, fructose and glucose may be the main sugars affecting the flavor of fruits [6,7]. Therefore, the excellent sweetness of both ‘Anna’ and ‘O’Neal’ may be caused by different types of sugar substances. This has been reported in other studies on blueberry cultivars [28]. In addition, we found seven carbohydrates were unregulated in both ‘Anna’ and ‘O’Neal’, including Echinacoside, L-Arabinitol, Catalpol, D-(−)-Fructose, Maltotriose, Coniferin and Gastrodin, indicating that these substances may be the common carbohydrates causing sweetness in blueberry cultivars.

KEGG enrichment of DAMs in the three comparison groups was also performed. For the One vs. Blu group, the metabolites were mainly related to flavor substances such as sesquiterpenoid and triterpenoid biosynthesis, terpenoid backbone biosynthesis and ubiquinone and other terpenoid-quinone biosynthesis, which were also found in the Ann vs. Blu group. We also found that the main pathways of metabolites of carbohydrates included starch and sucrose metabolism, fructose and mannose metabolism, amino sugar and nucleotide sugar metabolism and galactose metabolism in the One vs. Blu group. This was consistent with the diverse sugar substances. Comparatively, in the Ann vs. Blu group, the top five metabolites mainly included the metabolic pathways, phenylpropanoid biosynthesis, the biosynthesis of secondary metabolites, tyrosine metabolism and flavone and flavonol biosynthesis. As for the Duk vs. Blu group, the pathways of the metabolites of flavor substances were mainly involved in the galactose metabolism, fructose and mannose metabolism and monoterpenoid biosynthesis, which may be responsible for the synthesis of flavor or carbohydrate substances in ‘Duke’. This was also in accordance with there being no detection of sucrose in ‘Duke’ [26].

As the main role of carbohydrate substances in fresh food quality, a qRT-PCR verification of the expression of eight sugar metabolism genes in the blueberry cultivars was conducted. β-amylase plays a vital role in converting starch to maltose in plants [19] and we found a significant increase in D-(+)-maltose in the ‘O’Neal’ fruits. The sugar transporter protein, tonoplast monosaccharide transporter (TMT), is also involved in the accumulation of fruit sugars [37]. Here, *VcBAM* and *VcTMT* were both highly expressed in ‘Anna’, ‘Bluerain’ and ‘O’Neal’, which somewhat corresponded to the higher soluble sugar content and the detected D-(−)-Fructose, Sucrose and D-(+)-Glucose contents in these fruits. Both INV and SPS are main determinents in fruit sucrose content [38]. Here, the expression of *VcSPS* was at a lower level in ‘Anna’ and ‘Duke’, while *VcSPS1* was higher in ‘Duke’ and ‘O’Neal’, which may be the main reason for the higher sucrose levels in the ‘Duke’ and ‘O’Neal’ cultivars. The expression of *VcSS1* was found to be lower in ‘Anna’ and ‘O’Neal’, indicating its non-dominant role in the synthesis of sucrose. The principal enzymes involved in sugar accumulation during fruit maturation include INV and SPS [26,27]. They play important roles in fructose and glucose accumulation [31]. Soluble acid INV activity negatively correlates with the fruit sucrose concentration in blueberries [39]. In ‘Anna’, the expressions of *VcINV* and *VcNIN2* were at higher levels, while *VcINV9* was higher in both ‘Anna’ and ‘O’Neal’, indicating their vital roles in sugar substances, especially D-(−)-Fructose and D-(+)-Glucose, in these two blueberry cultivars. The differences in the higher levels of D-(−)-Fructose and D-(+)-Glucose in ‘Anna’ and the higher level of sucrose in ‘O’Neal’ may be partially responsible for their sweetness, and they rely on the expression of the related genes involved in sugar biosynthesis. 

Recently, the increased interest in the health benefits of blueberry fruits has promoted studies in horticulture and fruit quality traits, especially in terms of fruit flavor. The flavor of the blueberry fruit is largely determined by the content and variety of their particular volatile and nonvolatile compounds, as well as by their genetic factors. Further studies are needed to understand the biogenesis of blueberry flavor compounds using effective and high-throughput techniques that provide quantitative measurements of flavor attributes. Ferrᾶo et al. [40] used GC-MS to capture volatile compounds and linked them to genetic loci using a genome-wide association study to identify significant associations. This will be more useful in the future for breeding programs of blueberries to develop new cultivars with the desired flavor quality.

## 4. Materials and Methods

### 4.1. Plant Materials

The ripened fruits of eight southern highbush blueberry cultivars (‘Bluerain’, abbr. Blu; ‘O’Neal’, abbr. ONe; ‘Star’, abbr. Sta; ‘Camellia’, abbr. Cam; ‘Misty’, abbr. Mis; ‘Zhongzhi 3’, abbr. Zho; ‘Springhigh’, abbr. Spr; ‘Anna’, abbr. Ann) and three northern highbush blueberry cultivars (‘Brigitta’, abbr. Bri; ‘Darrow’, abbr. Dar; ‘Duke’, abbr. Duk) were used in this study. The five-year-old trees of these cultivars were field-grown at the test base of the Institute of Botany, Jiangsu Province, and the Chinese Academy of Sciences, Nanjing, China. Among them, the blueberry ‘Bluerain’ exhibited excellent fertility and adaptability at local conditions, while its taste is not as popular as the other ten cultivars. Then, fruit quality characteristics and metabolites of different blueberry cultivars with good flavor were compared and analyzed using ‘Bluerain’ as the control based on pedigree analysis (Table S1).

### 4.2. Analysis of the Appearance Quality of Different Blueberry Cultivars

The single-fruit weight of 30 fruits with the same ripeness and appearance was measured with a JY3002 balance of 0.01 g (Jiangke Co., Shanghai, China), and the diameter of single fruits was measured with an MNT-150 digital caliper of 0.01 mm (Meinaite Industrial Co Ltd., Shanghai, China). The fruit shape index of each cultivar was obtained based on calculation. The firmness (Newton, N) of small drupes near the peduncles was measured with a hand-held GY-4 fruit hardness tester (TOP Instrument Co., Hangzhou, China). The mean value of all tested traits was obtained using three replicates.

### 4.3. Analysis of the Fruit Flavor Quality Traits of Different Blueberry Cultivars

The content of soluble sugar in fruits was determined using the anthrone colorimetry method. The sugar was dehydrated to form aldehydes or hydroxymethyl aldehydes under the action of sulfuric acid, which could react with anthrone to form blue-green furfural derivatives at 620 nm. The soluble solids (BRIX) (SS) content in fruits was measured by a hand-held PAL-1 refractometer (Atago China Branch, Guangzhou, China). The titratable acidity (TA) content (citric acid) was determined by a ZD-2 automatic potentiometric titrator (Jinmai Instrument Co., Ltd. Shanghai, China) using a 0.1 M sodium hydroxide solution using the acid–base titration method [41]. The solid-acid ratio was calculated according to SS and TA data.

The anthocyanidin content was determined using 0.5 g of blueberry pericarp tissues and soaking them in 5 mL of 50% ethanol solution for 20 min at 35 °C. Anthocyanidin was spectrophotometrically assayed by measuring the optical density of the extraction solution at 510 nm. The extraction of the flavonoids was performed using 0.5 g of pulp and then refluxed with 50% alcohol. Then, after heating at 65 °C for 45 min, 2.5 mL of 95% ethanol solution was added and the optical density at 415 nm was measured. The total flavonoid content was then calculated based on the absorbance and the rutin concentration in the sample solution, determined from the standard working curve. The total polyphenols content was determined using the Hungarian forint method. After grinding, 0.5 g of fresh fruit was transferred into 5 mL of 50% ethanol solution. After ultrasonic treatment was carried out at 60 Hz at 35 °C for 20 min, 0.5 mL of forinol reagent and 2 mL of 7.5% sodium carbonate solution were added to determine the absorbance at 750 nm. The vitamin C content was tested in blueberries using a 2, 4-dinitrophenylhydrazine colorimetric method; the detection wavelength was 520 nm. Microsoft Excel 2010 was used for statistical processing and SPSS 25.0 was used for statistical significance analysis and correlation analysis.

### 4.4. Nontargeted LC-MS-Based Metabolic Profiling and Identification

The eleven fruit samples described above were used for metabolite extraction. A Dionex Ultimate 3000 RS UHPLC system fitted with a Q-Exactive quadru-pole-Orbitrap mass spectrometer equipped with a heated electrospray ionization (ESI) source (Thermo Fisher Scientific, Waltham, MA, USA) was used for non-targeted metabolomics research in this study. The experimental process mainly includes metabolite extraction, LC-MS/MS detection and data analysis. The original documents obtained by mass spectrometry detection were imported into the Compound Discoverer 3.1 software, and the qualitative and quantitative results of metabolites were obtained by spectrum processing and database searching. Data quality control was performed to ensure the accuracy of the data results and their reliability. Multivariate statistical analysis of metabolites, including principal component analysis (PCA) and partial least squares discriminant analysis (PLS-DA), was performed to reveal the differences in metabolic patterns among the groups. Hierarchical clustering (HCA) and metabolite correlation analysis were used to reveal the relationships among samples and between metabolites. Finally, functional and taxonomic annotation of the identified metabolites was carried out through metabolic pathways. The major databases included KEGG, HMDB, LIPID MAPS, etc., to understand the functional characteristics and classification of different metabolites. Three parameters, VIP, FC and *p*-value, were used to screen the differential metabolites. The threshold was set as VIP > 1.0, FC > 1.5 or FC < 0.667 and *p*-value < 0.05.

### 4.5. QRT-PCR Analysis

Total RNA was extracted using the RNA extraction kit (Bioteke Inc., Beijing, China) and then transcribed using a cDNA Synthesis Kit (CWBIO, Shanghai, China) from different fruit samples. Here, we detected the expression of eight key genes associated with sugar accumulation, including *VcBAM* (Genbank accession no. JQ911593), *VcINV* (GenBank accession no. MW383486), *VcSPS* (GenBank accession no. MT902322) and *VcTMT* (GenBank accession no. MT912540) as described by Nguyen et al. [26], and *VcSS1*, *VcSPS1*, *VcINV9* and *VcNIN2* as described by Wang et al. [27]. The primers for the genes used in this study are listed in Table S2. qRT-PCR on a qTOWER 2.2 qPCR instrument started with a denaturation step of 95 °C for 3 min, followed by 40 cycles of 95 °C for 10 s, 60 °C for 10 s and 72 °C for 15 s. Melting curves were derived using the following steps: 95 °C 15 s, 60 °C 30 s and 95 °C 15 s. The stably expressed blueberry reference gene *VcActin* (AY123769) was used as previously reported [27].

## 5. Conclusions

By collecting fresh food quality traits and biochemical data from different highbush blueberries with excellent flavor, we sought to generate the corresponding metabolites for improving blueberry flavor based on specific biochemical compounds. Fruit flavor analysis indicated that ‘Anna’ was the type with excellent sweetness, while having a lower degree of antioxidants, while the quality pattern in ‘Duke’ was to the contrary. ‘O’Neal’ was an excellent blueberry cultivar rich in both sweetness and antioxidants. We also identified certain pathways or biochemical compounds, especially in the sugar metabolism of these three cultivars, that were associated with increased consumer liking. The expression files of associated genes involved in the accumulation of sugar metabolites may partly elucidate the sweetness of different blueberry cultivars. The individual compounds detected in these cultivars may provide insight into a paradigm for the selection of desirable highbush blueberry cultivars in the future.

Limitations of the study: In this research, the integrative comparisons of flavor metabolites including sugars, organic acids and aroma substances on all cultivars were needed to verify the identified metabolites. Additionally, the GC-MS method needs to be further considered to analyze the flavor profiles in the three typical blueberry cultivars combined with the LC-MS/MS in this study. 

## Figures and Tables

**Table 1:** Statistics of appearance quality indices of highbush blueberry cultivars.

Cultivars	Transverse Diameter (mm)	Longitudinal Diameter (mm)	Fruit Shape Index *	Fruit Weight (g/per Berry)	Firmness (N)
Bluerain	12.83 ± 0.41 e	12.54 ± 0.48 cd	0.98 ± 0.04 a	0.94 ± 0.13 h	3.75 ± 0.49 a
O’Neal	15.36 ± 0.75 cd	12.36 ± 0.53 cd	0.81 ± 0.04 cd	1.47 ± 0.28 fg	2.64 ± 0.21 cd
Star	15.77 ± 0.96 bcd	12.79 ± 0.74 c	0.81 ± 0.04 cd	1.95 ± 0.28 cd	3.18 ± 0.19 b
Camellia	15.01 ± 0.80 d	11.22 ± 0.87 e	0.75 ± 0.05 ef	1.54 ± 0.35 ef	3.15 ± 0.33 b
Misty	16.08 ± 1.14 bc	12.45 ± 0.74 cd	0.78 ± 0.05 de	1.78 ± 0.39 cde	3.80 ± 0.27 a
Zhongzhi 3	15.71 ± 1.14 bcd	12.18 ± 0.45 cd	0.78 ± 0.04 cde	1.73 ± 0.32 def	3.03 ± 0.29 b
Springhigh	17.40 ± 1.01 a	14.20 ± 0.74 a	0.82 ± 0.05 bc	3.03 ± 0.49 a	2.72 ± 0.30 c
Anna	13.30 ± 0.93 e	11.30 ± 0.57 e	0.85 ± 0.04 b	1.23 ± 0.15 g	3.23 ± 0.17 b
Brigitta	17.90 ± 1.04 a	13.40 ± 0.69 b	0.75 ± 0.01 ef	2.60 ± 0.40 b	3.25 ± 0.15 b
Darrow	15.53 ± 0.86 cd	10.92 ± 0.73 e	0.70 ± 0.03 g	1.85 ± 0.29 cd	2.41 ± 0.18 d
Duke	16.51 ± 0.61 b	11.97 ± 0.49 d	0.73 ± 0.03 fg	2.06 ± 0.22 c	2.75 ± 0.21 c

Note: The different letters within the same column mean the significance at the 0.05 level. Data are represented as the mean + SD (standard deviation). * Fruit shape indexes were calculated as the ratio of longitudinal/transverse diameters.

**Table 2:** Statistics of nutritional quality indices of highbush blueberry cultivars.

Cultivar	Soluble Sugar (mg/g FW)	Titratable Acidity (TA) (%)	Soluble Solids (SS) (%)	SS/TA Ratio	Anthocyanin Content (mg/g FW)	Flavonoids Content (mg/g FW)	Total Polyphenols (mg/g FW)	VC Content (µg/mg FW)
Bluerain	151.45 ± 1.03 b	1.07 ± 0.02 a	18.42 ± 0.61 a	17.24 ± 0.29 de	1.08 ± 0.00	4.74 ± 0.07 c	0.28 ± 0.01 d	103.52 ± 7.18 a
O’Neal	143.50 ± 6.29 bc	0.27 ± 0.02	16.16 ± 0.06 b	60.96 ± 4.40 a	1.83 ± 0.00 c	4.89 ± 0.05 b	0.32 ± 0.01 b	88.58 ± 10.14 b
Star	126.06 ± 6.40 ef	0.79 ± 0.02 c	14.68 ± 0.08 cd	18.57 ± 0.44 d	1.03 ± 0.00	2.86 ± 0.07 g	0.27 ± 0.02 d	47.01 ± 1.24 e
Camellia	123.64 ± 1.03 fg	0.47 ± 0.02 g	14.92 ± 0.30 cd	31.84 ± 1.23 b	1.92 ± 0.00 b	4.14 ± 0.05 d	0.35 ± 0.01 a	104.42 ± 4.25 a
Misty	137.00 ± 1.67 cd	0.61 ± 0.02 e	14.30 ± 0.16 de	23.40 ± 0.67 c	1.15 ± 0.00 h	3.85 ± 0.07 e	0.35 ± 0.01 a	68.12 ± 1.79 c
Zhongzhi 3	129.26 ± 0.92 def	0.72 ± 0.02 d	12.72 ± 0.13 bf	17.76 ± 0.43 de	1.19 ± 0.01 g	2.85 ± 0.05 g	0.32 ± 0.01 b	49.46 ± 3.30 e
Springhigh	133.71 ± 1.93 de	0.44 ± 0.02 h	14.80 ± 0.07 cd	33.96 ± 1.36 b	1.61 ± 0.00 e	1.23 ± 0.07	0.30 ± 0.01 c	85.99 ± 4.23 b
Anna	172.01 ± 9.87 a	0.26 ± 0.02	16.50 ± 1.33 b	63.92 ± 4.42 a	0.72 ± 0.00	0.95 ± 0.07	0.23 ± 0.01 e	26.79 ± 1.29 f
Brigitta	120.84 ± 3.27 fg	0.86 ± 0.02 b	13.70 ± 0.61 e	15.96 ± 0.33 de	1.34 ± 0.00 f	2.66 ± 0.05 h	0.22 ± 0.00 e	58.08 ± 4.43 d
Darrow	126.36 ± 6.00 ef	1.03 ± 0.03 a	15.20 ± 0.10 c	14.71 ± 0.45 e	1.76 ± 0.00 d	3.69 ± 0.07 f	0.30 ± 0.01 c	119.52 ± 5.26 a
Duke	115.58 ± 4.34 g	0.53 ± 0.02 f	12.70 ± 0.55 f	23.80 ± 0.98 c	2.22 ± 0.01 a	5.56 ± 0.04 a	0.35 ± 0.01 a	108.13 ± 7.80 a

Note: The different letters within the same column mean significance at the 0.05 level. Data are represented as the mean + SD (standard deviation). FW, fresh weight.

## Data Availability

The data supporting the results in this study are included within the article.

## Supplementary Materials

The following supporting information can be downloaded at: https://www.mdpi.com/article/10.3390/plants12183200/s1, Table S1: Pedigree information of 11 highbush blueberry accessions; Table S2: Primer sequences of sugar metabolic genes in qRT-PCR for blueberry fruits; Table S3: Metabolite information of blueberry accessions under positive ion mode; Table S4: Metabolite information of blueberry accessions under negative ion mode.
