# Supplementary material for: Fruit Quality and Metabolomic Analyses of Fresh Food Accessions Provide Insights into the Key Carbohydrate Metabolism in Blueberry
Source: Plants (Basel). 2023 Sep 7;12(18):3200. doi: 10.3390/plants12183200 (PMC10535370; doi:10.3390/plants12183200)
Supplement: Supplementary file 1 [file plants-12-03200-s001.zip › Supplementary Table S1.pdf]

Table S1. Pedigree information of 11 highbush blueberry cultivars.

Cultivars	Type	Pedigree
Bluerain	SHB	No literature
O'Neal	SHB	Wolcott (Weymouth × (Stanley x Crabbe 4)) × Fla 4-15
Star	SHB	O'Neal ×FL80-31
Camellia	SHB	MS-122×MS-6（Darrow progeny）
Misty	SHB	Florida 67-1 (E-30 × Florida 61-7) × Avonblue (E-66 × Florida 1-3)
Zhongzhi3	SHB	No literature
Springhigh	SHB	No literature
Anna	SHB	No literature
Brigitta	NHB	Lateblue × Bluecrop
Darrow	NHB	(Wareham×Pioneer) × Bluecrop
Duke	NHB	(Ivanhoe×Earliblue) × (Berkeley×Earliblue) × (Coville×Atlantic)
