# Supplementary material for: Fruit Quality and Metabolomic Analyses of Fresh Food Accessions Provide Insights into the Key Carbohydrate Metabolism in Blueberry
Source: Plants (Basel). 2023 Sep 7;12(18):3200. doi: 10.3390/plants12183200 (PMC10535370; doi:10.3390/plants12183200)
Supplement: Supplementary file 1 [file plants-12-03200-s001.zip › Supplementary Table S2.pdf]

Table S2. Primer sequences of sugar metabolic genes in qRT-PCR for blueberry fruits.

Name	Forward primer (5' to 3')	Reverse primer (5' to 3')	Length/bp	References
VcBAM	GCTGGAACTCATTGGCACTACA	CCTGGTTGCTCTCCGTCTCT	170	Nguyen et al., 2021
VcINV	GTGCTCGTGAGATGCTTGCT	CCACCAACCAAGGAGGAATCTG	216	Nguyen et al., 2021
VcSPS	CGTTGAGGAGGAGGAAGCATAT	AAGACTGTGTTTCAGCCAAAGT	168	Nguyen et al., 2021
VcTMT	GTACAGAGTCCGCATCCTTCCT	TGTGGTGAGCAGTAGTGTCCTT	119	Nguyen et al., 2021
VcSPS1	CCAGGAGGAATCACAACCATGCG	TGCGGCGTATAGAGGCTGAGG	199	Wang et al., 2020
VcSS1	TCTCATCGTGACTCGGCTTCTCC	GCAACATCCTCGGTGAATGTCTCC	189	Wang et al., 2020
VcNIN2	AGCAAGAATGCGAGTGCCGATG	TGCTTCGAGAGGCTGTTGTGAAC	139	Wang et al., 2020
VcINV9	GTGTAGCCGAGCCTATGTTCAGTG	CAGTGGAGGAAGTGGAGAAGTTGC	102	Wang et al., 2020
VcActin	CTTGCATCCCTCAGCACCT	TCCTGTGGACAATGGATG	171	Wang et al., 2020
